# Statins and Cardiovascular Disease Outcomes in Chronic Kidney Disease: Reaffirmation vs. Repudiation

**DOI:** 10.3390/ijerph15122733

**Published:** 2018-12-04

**Authors:** Chamberlain I. Obialo, Elizabeth O. Ofili, Keith C. Norris

**Affiliations:** 1 Department of Medicine, Morehouse School of Medicine, Atlanta, GA 30310-1495, USA; eofili@msm.edu; 2 Department of Medicine, David Geffen School of Medicine, University of California Los Angeles, Los Angeles, CA 90024, USA; KCNorris@mednet.ucla.edu

**Keywords:** Statins, chronic kidney disease, cardiovascular disease

## Abstract

Cardiovascular disease (CVD) burden is several-fold higher in patients with chronic kidney disease (CKD). Although statins have been shown to provide significant CVD benefits in both the general population and patients with CKD, this has not translated into survival advantage in patients with advanced CKD or on dialysis. It has been reported that CVD risk continues to escalate as CKD progresses to end-stage kidney disease (ESKD); however, the CVD risk reduction by statins appears to decline as patients’ progress from the early to later stages of CKD. Statins have also been associated with a higher incidence of stroke in ESKD patients. Thus, the CVD benefits of statins in ESKD remain questionable.

## 1. Introduction

The prevalence of cardiovascular disease (CVD) has been reported to be 10-fold higher in patients with chronic kidney disease (CKD) than in the general population [1]. This CVD risk progressively increases as CKD progresses to end-stage kidney disease (ESKD) [2]. Ultimately, CVD accounts for about 50% of ESKD mortality [2,3]. In addition to the known traditional risk factors for CVD, such as age, hypertension, diabetes mellitus, and hyperlipidemia, patients with CKD have other nontraditional risk factors. These factors include anemia, albuminuria, bone and mineral disorders, inflammation [4,5], and, more recently, fibroblast growth factor 23 (FGF-23) [6,7]. It has also been suggested that CKD per se is a risk factor for the development of CVD [8]. Overall, there are strong similarities between risk markers of CKD and CVD. The prevalence of these associations has been well documented [9,10].

It has been shown that as CKD progresses to ESKD, the relative impact of atherosclerotic burden declines while non-atherosclerotic conditions escalate. Unlike the general population, in whom advanced atherosclerosis manifest as fibro-atheromatous intimal lesions, calcific medial lesions are classically seen in ESKD [11]. Thus the beneficial effects on CVD of lowering low-density lipoprotein (LDL) cholesterol may not fully apply to ESKD patients.

## 2. Lipid Lowering in the General Population and in CKD

Lipid-lowering drugs such as 3-hydroxyl-3-methylglutaryl CoA reductase inhibitors (statins) have been overwhelmingly demonstrated to reduce CVD morbidity and mortality by 23–31% in the general population [12,13,14,15,16,17]; however, patients with CKD were generally excluded from these studies. One of the exclusion criteria in some of those trials is serum creatinine ≥ 1.3–2.0 mg/dL [13,14,15] or just the presence of renal disease [16]. Hence, the initial reports on the beneficial effects of statins in CKD were derived mainly from post hoc analysis of pooled data [18,19]. The Aggressive Lipid-Lowering Initiation Abates New Cardiac Events (ALLIANCE) Study [18] showed that atorvastatin use was associated with a 28% risk reduction in CKD patients, while the Pravastatin Pooling Project [19], which used pooled data from three randomized trials of pravastatin vs. placebo [13,16,17], reported that pravastatin significantly reduced cardiovascular events in people with moderate CKD known to have coronary heart disease (CHD). 

## 3. Lipid Lowering in CKD/ESKD Patients

Patients with CKD differ significantly from the general population in their lipid patterns. Hypertriglyceridemia, reduced high-density lipoprotein (HDL) cholesterol, and elevated small dense LDL particles are frequently observed prevalent patterns in CKD [20]. Patients on peritoneal dialysis usually have high LDL cholesterol, which mimics the nephrotic syndrome pattern because of the marked protein loss with the dialysis. In view of these variable lipid anomalies in CKD/ESKD, the beneficial effects on CVD of LDL reduction with statins remains debatable in ESKD. Contrary to the graded positive associations seen in the general population, a U-shaped association between cholesterol levels and all-cause mortality is seen in hemodialysis patients [21].

As a result of the exclusion of patients with advanced CKD and ESKD from major cholesterol trials, initial reports on mortality benefits of statins in this cohort were based on large national databases, such as the United States Renal Data System Dialysis Morbidity and Mortality Study: Wave 2 [22]. This initial report noted that statin use in the dialysis population was associated with a 32% risk reduction in CVD mortality. A post hoc analysis of the ALLIANCE Study [18], which involved 579 patients with CKD, CHD, and dyslipidemia, compared atorvastatin therapy with usual care. The authors reported that atorvastatin reduced the relative risk of their primary outcome (time of first cardiovascular event) by 28% in CKD patients. Considering these initial positive reports [18,19,22], an urgent need for a prospective statin trial in ESKD became apparent. The Assessment of Lescol in Renal Transplant (ALERT) Study [23] compared use of fluvastatin with placebo in post–renal transplant patients with mean glomerular filtration rate (GFR) of 60 mL/min. The authors noted that LDL cholesterol was lowered by 32%, but CVD risk reduction was not significantly lower. The authors concluded that the cardiac event rates were lower and that the study had insufficient power to detect a significant reduction in their primary endpoint of cardiac death, nonfatal myocardial infarction, or coronary intervention procedure. Similarly, the Prevention of Renal and Vascular End Stage Disease–Intervention Trial (PREVENT-IT) [24] was unable to demonstrate a CVD morbidity/mortality benefit of pravastatin over placebo in patients with early CKD who were followed over a 46-month period.

The first published multicenter randomized study on the effect of statins in hemodialysis patients at risk for CVD and death was the Deutsche Diabetes Dialyse Studie (4D study) [25]. The study involved 1255 patients followed over a 4-year period and compared atorvastatin therapy to placebo. They observed that atorvastatin reduced LDL by 30–50 points but had no statistically significant effect on the composite primary endpoint of cardiovascular death, nonfatal myocardial infarction, and stroke in patients with diabetes receiving dialysis. However, the relative risk of fatal stroke was significantly higher in patients receiving atorvastatin (Relative Risk (RR) 2.03, *p* = 0.04).

The second randomized published trial in dialysis patients was the rosuvastatin and Cardiovascular Events in Patients Undergoing Hemodialysis (AURORA) Study [26]. This study included 2776 ESKD patients with and without diabetes followed for about 4 years. The authors also concluded that although LDL cholesterol was significantly reduced by rosuvastatin, it had no significant effect on their composite primary endpoint of Cardiovascular (CV) death, nonfatal myocardial infarction, or nonfatal stroke. As was observed in the 4D trial, the incidence of nonfatal stroke was higher in the statin group.

The third trial involving dialysis patients was the Study of Heart and Renal Protection (SHARP) trial [27], but unlike the previous two studies, this study included 6247 CKD patients not on dialysis and 3023 ESKD patients on dialysis who were followed for about 5 years. The study reported that compared to placebo, ezetimibe plus simvastatin in combination produced a 17% proportional risk reduction in their pre-specified outcomes of nonfatal myocardial infarction, coronary death, non-hemorrhagic stroke, or any arterial revascularization procedure. The SHARP authors also noted that their study did not have sufficient power to assess the CVD outcomes separately between the dialysis and non-dialysis patients. However, they concluded that there was no significant heterogeneity between dialysis and non-dialysis patients (meaning there was no evidence that the proportional effects of the intervention differed between the two groups). Unlike the 4D and AURORA studies, the SHARP trial has been extensively criticized because of its design. It combined CKD and ESKD patients, and about a third of the non-dialysis patients at baseline began dialysis within the first year of the study. It also included soft CVD endpoints such as arterial revascularization and failed to examine the effect of ezetimibe alone compared to the combination of ezetimibe plus simvastatin. In a post hoc analysis of over 18,000 patients, the combination of ezetimibe plus simvastatin was found to be more effective than monotherapy in patients with CKD (GFR 30–60 mL/min/1.73 m^2^) [28]. Hence the role of ezetimibe in the SHARP findings remains unknown.

The beneficial effects on CVD observed in the SHARP trial were more pronounced in the CKD patients and appeared to decline as CKD progressed to ESKD. A post hoc analysis of SHARP showed that CVD event rate prevention decreased from 2.5% to 1.5% at GFR 30–60 mL/min/1.73 m^2^ in dialysis patients, while the risk of an event increased by 7.9%, 10.2%, 10.9%, and 15% as GFR declined to 30–60, 15–30, ˂15, and dialysis, respectively [29,30]. Similarly, two major meta-analyses [31,32] reported that statins reduced relative risk of all-cause mortality in CKD but not in persons receiving dialysis. Finally, the Cholesterol Treatment Trialists’ (CTT) collaboration, which involved 28 trials with over 183,000 patients, noted that the relative reductions in major vascular events observed with statin-based treatment became smaller as GFR declined, with little evidence of benefit in patients on dialysis, and in addition, the risk of stroke was higher with LDL cholesterol lowering in dialysis patients [32]. The value of lowering cholesterol in ESKD patients has been called into question because of the U-shaped association with mortality in ESKD [21]. Moreover, hypocholesterolemia has been shown to be a negative acute phase reactant that portends high mortality in ESKD [21,33] and a predictor of mortality in acute kidney injury [34].

Apart from the stroke risk, it has been shown that statins may promote vascular calcifications by inhibition of vitamin K synthesis [35]. Increased coronary artery calcification is known to predict mortality in ESKD [36]. Current guidelines from both the American College of Cardiology/American Heart Association (ACC/AHA) [37] and Kidney Disease Improving Global Outcomes (KDIGO) [38] do not recommend initiation or continuation of statin therapy in individuals on maintenance hemodialysis. There are no data on whether or not statins are effective for secondary CVD prevention in patients on dialysis who get their first cardiac event. KDIGO, however, suggests that statins be continued in dialysis patients who are already receiving it at the time of dialysis initiation.

## 4. Inflammation and Cholesterol in ESKD

As mentioned earlier, the relationship between ESKD mortality and cholesterol level is that of a U-shaped curve [21,39]. The phenomenon of “reverse causality” has been used to explain this relationship [40,41]. In that sense, CKD and its associated comorbid conditions cause lower cholesterol levels and increased risk of death. It has been postulated that this inverse relationship between cholesterol and ESKD mortality may also be due to the cholesterol-lowering effects of systemic inflammation and malnutrition, which are prevalent in dialysis patients, and not to a protective effect of high cholesterol. Therefore, hypercholesterolemia should be treated in this cohort [39]. Inflammation is known to play a role in the pathobiology of CVD [42], but it is debatable whether chronic inflammation as seen in the dialysis population influences the relationship between LDL cholesterol and CVD. An experimental study utilizing vascular smooth muscle cells has demonstrated that inflammation may reduce the efficacy of statin therapy by enhancing cholesterol synthesis and intracellular lipid accumulation [43]. However, there was no evidence for statin resistance in all three major statin trials in the ESKD population, as LDL cholesterol levels were appropriately lowered in all treatment arms of the studies [25,26,27]. Some investigators have reported that statin therapy was more effective in the presence of inflammation and that patients who achieved lower C-reactive protein (CRP) levels had better CVD outcomes [44,45]. The relationship between inflammation and LDL cholesterol was recently examined in a post hoc analysis of the SHARP data [46]. The authors reported that the efficacy of lowering LDL cholesterol with simvastatin/ezetimibe on major vascular events was similar irrespective of the baseline CRP concentration, thus there was no evidence that the relative beneficial effects of reducing LDL cholesterol depended on plasma CRP concentrations. Given these findings, it seems reasonable to assume that the presence of inflammation should not influence the decision to treat hypercholesterolemia in CKD/ESKD. 

## 5. Conclusions/Summary

Statins have been overwhelmingly demonstrated to provide CVD protection in the general population and in early CKD. The CVD beneficial effect of statins in CKD appears analogous to that seen in the general population, but its benefit in advanced CKD or ESKD remains questionable. Cardiovascular disease prevalence is much higher in CKD/ESKD patients than in the general population, but the relative atherosclerotic burden declines as CKD progresses toward ESKD, while non-atherosclerotic conditions escalate. The value of lowering LDL cholesterol in dialysis patients is less clear and may be associated with higher risk of stroke. Mortality in ESKD involves an interplay of multiple factors beyond reduction of LDL cholesterol. The complex interrelationship between inflammation, cholesterol level, and mortality in ESKD warrants further studies. 

We generally prescribe statins to all our CKD patients when indicated, but statin therapy for our dialysis patients is prescribed with caution and as part of a shared decision-making process with the patients and their cardiologists. Other non-statin lipid-lowering drugs have not been extensively studied in the CKD/ESKD population because of the concern for worsening CKD. The Fenofibrate Intervention and Event Lowering in Diabetes (FIELD) study [47] noted that compared to placebo, fenofibrate reduced total CV events in patients with moderate CKD (eGFR 30–59 mL/min/1.73 m^2^) but ESKD rates were similar. A recent post hoc analysis of 2636 participants in the fenofibrate arm of the Action to Control Cardiovascular Risk in Diabetes (ACCORD) trial [48] also noted that compared to placebo, rates of albuminuria were lower while eGFR decline was slower, but there was no difference in the incidence of CKD. More randomized studies will be needed to fully clarify the risk/benefit effect of fibrates in the CKD/ESKD population. 

The impact of malnutrition/inflammation and reduction of LDL cholesterol on CV mortality in the dialysis population requires further exploration. Future studies on biomarkers that may identify the subgroup of dialysis patients who are likely to benefit from LDL cholesterol lowering should be of interest. In addition, a study on the role of newer agents such as proprotein convertase subtilisin/kexin 9 (PCSK 9) inhibitors in ESKD morbidity/mortality is warranted.
